# Remarkable repellency of *Ligusticum sinense* (Umbelliferae), a herbal alternative against laboratory populations of *Anopheles minimus* and *Aedes aegypti* (Diptera: Culicidae)

**DOI:** 10.1186/s12936-015-0816-y

**Published:** 2015-08-07

**Authors:** Rukpong Sanghong, Anuluck Junkum, Udom Chaithong, Atchariya Jitpakdi, Doungrat Riyong, Benjawan Tuetun, Daruna Champakaew, Jitrawadee Intirach, Roongtawan Muangmoon, Arpaporn Chansang, Benjawan Pitasawat

**Affiliations:** Department of Parasitology, Faculty of Medicine, Chiang Mai University, Chiang Mai, 50200 Thailand; Department of Food Industry and Service, School of Culinary Arts, Suan Dusit Rajabhat University Lampang, Lampang, 52000 Thailand

**Keywords:** *Ligusticumsinense*, Repellents, Mosquitoes, *Anopheles minimus*, *Aedes aegypti*

## Abstract

**Background:**

For personal protection against mosquito bites, user-friendly natural repellents, particularly from plant origin, are considered as a potential alternative to applications currently based on synthetics such as DEET, the standard chemical repellent. This study was carried out in Thailand to evaluate the repellency of *Ligusticumsinense* hexane extract (LHE) against laboratory *Anopheles minimus* and *Aedes aegypti*, the primary vectors of malaria and dengue fever, respectively.

**Methods:**

Repellent testing of 25% LHE against the two target mosquitoes; *An. minimus* and *Ae. aegypti*, was performed and compared to the standard repellent, DEET, with the assistance of six human volunteers of either sex under laboratory conditions. The physical and biological stability of LHE also was determined after keeping it in conditions that varied in temperature and storage time. Finally, LHE was analysed chemically using the qualitative GC/MS technique in order to demonstrate a profile of chemical constituents.

**Results:**

Ethanol preparations of LHE, with and without 5% vanillin, demonstrated a remarkably effective performance when compared to DEET in repelling both *An. minimus* and *Ae. aegypti*. While 25% LHE alone provided median complete-protection times against *An. minimus* and *Ae. aegypti* of 11.5 (9.0–14.0) hours and 6.5 (5.5–9.5) hours, respectively, the addition of 5% vanillin increased those times to 12.5 (9.0–16.0) hours and 11.0 (7.0–13.5) hours, respectively. Correspondingly, vanillin added to 25% DEET also extended the protection times from 11.5 (10.5–15.0) hours to 14.25 (11.0–18.0) hours and 8.0 (5.0–9.5) hours to 8.75 (7.5–11.0) hours against *An. minimus* and *Ae. aegypti*, respectively. No local skin reaction such as rash, swelling or irritation was observed during the study period. Although LHE samples kept at ambient temperature (21–35°C), and 45°C for 1, 2 and 3 months, demonstrated similar physical characteristics, such as similar viscosity and a pleasant odour, to those that were fresh and stored at 4°C, their colour changed from light- to dark-brown. Interestingly, repellency against *Ae. aegypti* of stored LHE was presented for a period of at least 3 months, with insignificantly varied efficacy. Chemical analysis revealed that the main components of LHE were 3-*N*-butylphthalide (31.46%), 2, 5-dimethylpyridine (21.94%) and linoleic acid (16.41%), constituting 69.81% of all the extract composition.

**Conclusions:**

LHE with proven repellent efficacy, no side effects on the skin, and a rather stable state when kept in varied conditions is considered to be a potential candidate for developing a new natural alternative to DEET, or an additional weapon for integrated vector control when used together with other chemicals/measures.

## Background

Over half the world’s population is currently at risk from vector-borne diseases, particularly malaria and dengue fever. In 2012, an estimated 1.2 billion people living mostly in African (47%) and Southeast Asian (37%) regions were at high risk of contracting malaria, and 207 million actual cases produced 627,000 deaths worldwide [1]. Among 500 species of *Anopheles* mosquitoes described globally, more than 50 can transmit malaria from the bite of an infected female mosquito [2, 3]. One of the strategies for preventing and controlling this disease is to emphasize on mosquito management. *Anopheles minimus* is a main malaria vector in the hilly, forested regions of mainland Southeast Asia, including Thailand, and also the main target of vector control in this area [4–6]. In the last 10 years, the incidence of malaria in Thailand has been declining substantially, due to increased funding for malaria control, intensive vector control measures, such as indoor residual spraying (IRS) with chemical insecticides, and improved access to personal protection measures [7, 8]. However, serious challenges remain; particularly in the growing migrant populations from malaria-endemic areas located along the western and eastern forested borders with Myanmar and Cambodia, respectively. Furthermore, once the Asean Economic Community (AEC) comes into force by late 2015, Thailand and the other Asean countries will face more environmental and health problems, due to the free flow of labour that could lead to crowded living conditions and disease outbreaks.

Dengue and dengue haemorrhagic fever also are dangerous diseases, and a growing threat to global health. Approximately 2.5 billion people around the world; mostly in the tropics, such as urban regions of Southeast Asia, the Americas, Africa and the Pacific area are currently at risk of dengue infections. Although the risk of dengue is higher usually in urban rather than non-urban regions, dengue infections in rural communities have been reported increasingly, particularly in Thailand, where the incidence rate is higher in rural (102.2 per 100,000) than urban areas (95.4 per 100,000) [9–11]. Among 950 species of *Aedes* mosquitoes registered worldwide; *Aedes aegypti* and *Aedes albopictus* transmit dengue [12, 13].

Despite decades of organized control over diseases and vectors, malaria and dengue remain major public health problems causing significant mortality and morbidity together with great financial loss in most tropical and subtropical regions of the world [1, 8]. Apart from the absence of a prophylactic vaccine or specific therapeutic treatment for malaria and dengue, current control of these communicable diseases is more difficult, due to the increased resistance of mosquito populations and pathogens, particularly malarial parasites, to synthetic insecticides and chemotherapeutic drugs, respectively [14–16]. Consequently, individual responsibility such as personal protection from mosquito attacks, including repellent usage, has been projected as an important weapon for preventing vector-borne diseases. Through their minimizing man-mosquito contact results in reducing the risk of disease transmission; therefore, repellents already have been accepted as part of an overall integrated mosquito-borne disease control programme [17].

Currently, there are a variety of synthetic and botanical-derived chemicals proven to repel mosquitoes. However, few repellents are considered effective and safe enough to be applied repeatedly to the skin. The best-known synthetic chemical is *N,N*-diethyl-3-methylbenzamide (DEET), which is accepted as the most effective broad spectrum insect repellent component, with a long lasting effect on mosquitoes and other biting arthropods. At present, DEET is the main active ingredient in most repellents commercially available to consumers throughout the world under a variety of brand names, as 5–100% concentrations in various formulations are applied to the skin and clothing [18–20]. Despite being considered safe, DEET is recommended for use with caution because of its damaging effects on plastic and synthetic fabric as well as toxic reactions, such as dermatitis, allergy, and neurologic and cardiovascular side effects, which have been described mainly after misapplication [18, 21, 22]. Repellents of plant origin are incorporated generally as active ingredients with one or more essential oils of citronella, eucalyptus, geraniol, peppermint, soybean or sedarwood [20, 23]. Even though botanical-based repellents are marketed nowadays worldwide under different brands with multiple formulations, and the demand is increasing dramatically; their crucial disadvantages are limited efficacy and protective duration as well as high cost [23, 24]. It is, therefore, necessary to evaluate new bioactive substances, particularly herbal-based products with high potential for personal protection against mosquitoes and mosquito-borne diseases.

In a previous screening program for new repellents from fifteen medicinal plants, the hexane extract of *Ligusticum sinense* rhizomes possessed the most promising repellency against laboratory-reared *Ae. aegypti*, with a median complete-protection time of 6.5 (5.0–8.0) hours [25], which exceeded by far the minimum requirement of 2 h established by the National Institute of Health, Thailand. In continued research to identify and develop new botanical repellents, *L. sinense* hexane extract (LHE) was evaluated against two mosquito vectors, *An. minimus* and *Ae. aegypti*. Also, assessment of physical and biological performances, and GC/MS characterization were performed to show stability and profile, respectively, of the chemicals comprised in LHE.

## Methods

### Plant materials and extractions

Rhizomes of *L. sinense* were obtained commercially from a herbal supplier in Chiang Mai province, northern Thailand. Taxonomic identification of this plant material was performed by Miss Wannaree Charoensup, a scientist at the Department of Pharmaceutical Science, Faculty of Pharmacy, Chiang Mai University (CMU), Chiang Mai 50200, Thailand. A voucher specimen was deposited at the Department of Parasitology, Faculty of Medicine, CMU under accession number PARA-LI-001/1. After air drying at ambient temperature (30 ± 5°C) under indoor conditions, *L. sinense* rhizomes were pulverized to a fine powder with the aid of an electrical blender. Half a kilogram of air-dried powdered rhizomes was extracted successively by maceration with 5 l of hexane at room temperature (27 ± 5°C) for at least 7 days until all the extractable components were exhausted. After vacuum filtration through a Bücher funnel with Whatman No. 1 filter paper, the solvent in combined filtrates was removed on a rotary evaporator (EYELA, Japan) at 60°C until it had evaporated completely. The residues were then lyophilized to afford a brown semisolid extract with a characteristic aromatic odour, and subsequently kept at 4°C until required for phytocomponent analysis and repellent evaluation.

### Mosquitoes

The mosquitoes tested in this study were composed of free-mating laboratory *An. minimus* sensu stricto (formerly *An. minimus* A) and *Ae. aegypti*, which are the principal vectors of malaria and dengue fever, respectively, in Thailand. The former was obtained originally from the Office of Vector Borne Diseases Control, Department of Communicable Disease Control, Ministry of Public Health, Chiang Mai province in 1997; and the latter originated from field larvae collected from clean stagnant water at various places in Chiang Mai province in 1995. These mosquitoes were colonized and maintained continuously from the dates obtained without exposure to any insecticides or pathogens for several generations in standard conditions (27 ± 2°C, 85 ± 5% RH and 14:10 h light/dark photoperiod cycle) in an insectary at the Department of Parasitology, Faculty of Medicine, CMU. Adults were fed ad libitum with 10% sucrose and 10% v/v multivitamin syrup. Blood meals were provided periodically to female mosquitoes for egg maturation from restrained albino rats kept in the breeding cages. Unfed female mosquitoes (5–7 days old), which derived from these mosquito colonies, were used for investigations on repellent efficacy. Prior to testing, female mosquitoes were starved by only accessing water for 12 h in order to stimulate blood feeding during the repellent experiments.

### Human volunteers

Six healthy volunteers of either sex (aged 21–35 years old; weight 44–93 kg) were recruited into this study from CMU graduate students, with no history of allergic reactions to arthropod bites, stings or repellents or dermatological disease. The participants were interviewed and advised fully on the purposes and methodology of this study, probable discomforts from exposure to test substances and mosquito bites, and remedial arrangements, before signing an informed consent form under protocol PAR-11-808-EX, approved by the Research Ethics Committee of the Faculty of Medicine, CMU. The volunteers also were advised to avoid alcohol and any fragrant products such as perfume, cologne, deodorant and lotion during the entire study period.

### Evaluation on repellent activity

Repellent evaluation of LHE was performed with six human volunteers (3 females and 3 males) against two target mosquitoes, *An. minimus* and *Ae. aegypti*, under laboratory conditions. DEET, the standard synthetic repellent, was used as a positive control and benchmark for comparing repellent efficacy. The test solution of LHE or DEET was prepared at the concentration of 25% by dissolving in absolute ethanol with and without 5% vanillin. The experiments were conducted inside a standard mosquito cage (30 × 30 × 30 cm), with the human-bait method modified from the WHO standard procedure [26]. The timing of the test periods depended on whether the target mosquitoes were night or day biters. As each mosquito species has preferences in biting time; night-biting *An. minimus* was tested from 18.00–08.00 h, while *Ae. aegypti*, the day biter, was tested between 06.00 and 18.00 h.

A total of 250 blood-starved female mosquitoes were selected randomly, transferred to each experimental cage and left to acclimatize for 1 h. Volunteer arms were washed with distilled water, allowed to air dry, and then rubber gloves were worn over the hands. The volunteer’s entire forearm was wrapped in a plastic sleeve, with a 3 × 10 cm open window on the ventral part providing exposure to mosquitoes. Approximately 0.1 ml of test solution was applied to the exposed skin on one forearm of each volunteer with the help of a pipette. The other forearm acted as a control by being treated with absolute ethanol (with and without 5% vanillin), and using a similar protocol to that for the tested arm. After air drying for 1 min, the control arm was inserted into an experimental cage for 3 min in order to make comparative checks and determine mosquito biting activity. If at least 10 mosquitoes landed on the control arm, the repellency test was then continued by exposing the treated forearm in a similar manner. The control and test arms were interchanged regularly to test for readiness of the mosquitoes to bite, and prevent any bias. The complete protection time was recorded after exposing the treated forearm for 3 min at 30-min intervals until either the first two bites occurred in a single exposure period or one bite occurred in each of two consecutive ones. Each test was repeated on each of the six volunteers with a new batch of mosquitoes on different days, and no volunteer tested more than 1 sample per day. Therefore, each sample could be tested twice on each subject and there were 12 replicates for each sample test. Randomization was used to assign the order of tests and treatment of volunteers, who were blinded to the repellent applied. Skin irritation, hot sensation, and other undesirable effects were observed from each experiment.

### Testing the physical and biological stability of LHE

Physical and biological stability of LHE were assessed by observing physical changes and determining persistence of repellent activity, respectively, after being kept in conditions that varied in temperature and storage time. The test for repellent activity followed a modification of the WHO standard method [26], as described previously. For this step, LHE samples were kept at various temperatures (4°C, ambient temperature and 45°C) for different durations (1, 2 and 3 months). Subsequently, they were observed physically and tested for repellency against *Ae. aegypti*. The results obtained were then compared to earlier data on fresh preparation for physical characteristics and protection times. The repellent test was conducted twice on each of the six human volunteers.

### Gas chromatography–mass spectrometry (GC/MS) analysis

GC/MS analysis of LHE was performed using a Hewlett-Packard 6890 gas chromatographer (Agilent Technologies; Germany) equipped with a split-splitless injector and column DB-5 MS (30 m × 0.25 mm × 0.25 µm film thickness) directly coupled to a quadrupole mass selective detector, MSD 5973 inert (Agilent Technologies; USA). The operating conditions were programmed as follows: helium was used as the carrier gas at a constant flow rate of 1.0 ml/min. One µl of LHE sample was injected neat with a split ratio of 10:1. The injector temperature was maintained at 250°C and the oven temperature programmed from 40°C (isothermal for 5 min), increasing at 5°C/min until reaching 7 min isothermal at 280°C. For GC/MS detection, an electron ionization system was operated in electron impact mode with ionization energy of 70 eV. The ion source and quadrupole temperature were set at 230 and 150°C, respectively. Electron impact spectra in positive ionization mode were acquired between 20 and 350 *m*/*z*. Chemical components of LHE were identified by comparing with standards by spiking, and on the basis of their mass spectral fragmentation using the Wiley 7n.1 spectral library. Percentage of the identified compound was computed from a Total ion chromatogram (TIC).

### Data processing and analysis

The median complete-protection time was used as a standard criterion for the repellency of the tested substances against *An. minimus* and *Ae. aegypti*. The effect of vanillin in prolonging the protection time of ethanolic preparations of LHE and DEET were analyzed using the Mann–Whitney U test. Significant differences in repellent efficacy among the test samples were inferred by non-overlapping confidence intervals around the average protection time of each sample.

## Results

### Repellent activity

Both mosquito species, *An. minimus* and *Ae. aegypti* showed high avidity with no difference in their biting tendency on the controls. Results in Table 1 demonstrated that *Ae. aegypti* proved to be more tolerant than *An. minimus* towards both LHE and DEET. The median complete-protection times against *Ae. aegypti* of all repellent samples, with and without 5% vanillin, including LHE, DEET, LHEv, DEETv, were shorter than those against *An. minimus*. Ethanol preparations of LHE, with and without 5% vanillin, demonstrated a remarkably effective performance that was comparable to DEET in repelling both tested mosquito species. While 25% LHE alone provided median complete-protection times against *An. minimus* and *Ae. aegypti* of 11.5 (9.0–14.0) hours and 6.5 (5.5–9.5) hours, respectively, the incorporation of 5% vanillin increased LHE repellency against *An. minimus* and *Ae. aegypti* with prolonged median complete-protection times of 12.5 (9.0–16.0) hours and 11.0 (7.0–13.5) hours, respectively. Correspondingly, vanillin also extended the protection times of 25% DEET against *An. minimus* and *Ae. aegypti* from 11.5 (10.5–15.0) hours to 14.25 (11.0–18.0) hours and 8.0 (5.0–9.5) hours to 8.75 (7.5–11.0) hours, respectively. However, there was no significant difference in the effect of vanillin in prolonging the protection by LHE and DEET products against *An. minimus* and *Ae. aegypti* (p > 0.05).

**Table 1:** Repellent activity of the ethanolic preparations of LHE and DEET, with and without 5% vanillin, against *Anopheles minimus* and *Aedes aegypti*

Repellent sample	Median complete-protection time (range, hours)^a^	
	*An. minimus*	*Ae. aegypti*
25% LHE	11.5 (9.0–14.0)	6.5 (5.5–9.5)
25% LHE + 5% vanillin (25% LHEv)	12.5 (9.0–16.0)	11.0 (7.0–13.5)
25% DEET	11.5 (10.5–15.0)	8.0 (5.0–9.5)
25% DEET + 5% vanillin (25% DEETv)	14.25 (11.0–18.0)	8.75 (7.5–11.0)

^a^There were 12 replicates in each test (6 volunteers).

### Physical and biological stability

The physical and biological performances of LHE samples, determined after storage under different conditions that varied in temperature [4°C, ambient temperature (21–35°C) and 45°C] and duration (1, 2 and 3 months), were slightly different (Table 2). Some changes in physical characteristics and varying degrees of repellency were recorded among stored LHE. For physical observation, the appearance and odour of all stored LHE samples were similar to those of the fresh sample, with a viscous and pleasant aromatic fragrance; whereas the colour of samples kept at ambient temperature and 45°C for 1, 2 and 3 months changed from light- to dark- brown. The repellent activity against *Ae. aegypti* of the stored LHE samples was present for a period of at least 3 months, with insignificantly varied efficacy (3.5–8.0 h). Apart from the LHE samples stored for 1 month, most samples kept at each temperature for 2 and 3 months exhibited slightly lower repellency (3.5–6.5 h) than the fresh sample (5.0–8.0 h).

**Table 2:** Physical characteristics and repellency against *Aedes aegypti* of the fresh and stored samples of LHE

LHE samples (temperature/duration)	Physical characteristics			Median complete–protection time (range, hours)^a^
	Appearance	Colour	Odour	
Fresh sample	Viscous	Light-brown	Aromatic	6.5 (5.0–8.0)
Stored sample				
4°C				
1 month	Viscous	Light-brown	Aromatic	7.5 (5.0–9.0)
2 months	Viscous	Light-brown	Aromatic	5.25 (3.5–6.5)
3 months	Viscous	Light-brown	Aromatic	4.25 (3.0–6.5)
Ambient temperature (21–35°C)				
1 month	Viscous	Dark-brown	Aromatic	7.25 (5.0–10.5)
2 months	Viscous	Dark-brown	Aromatic	6.5 (3.5–8.0)
3 months	Viscous	Dark-brown	Aromatic	5.5 (3.0–6.5)
45°C				
1 month	Viscous	Dark-brown	Aromatic	8.0 (4.5–8.5)
2 months	Viscous	Dark-brown	Aromatic	4.25 (3.0–6.5)
3 months	Viscous	Dark-brown	Aromatic	3.5 (2.5–5.5)

^a^There were 12 replicates in each test (6 volunteers).

### Chemical composition

**Fig. 1:**
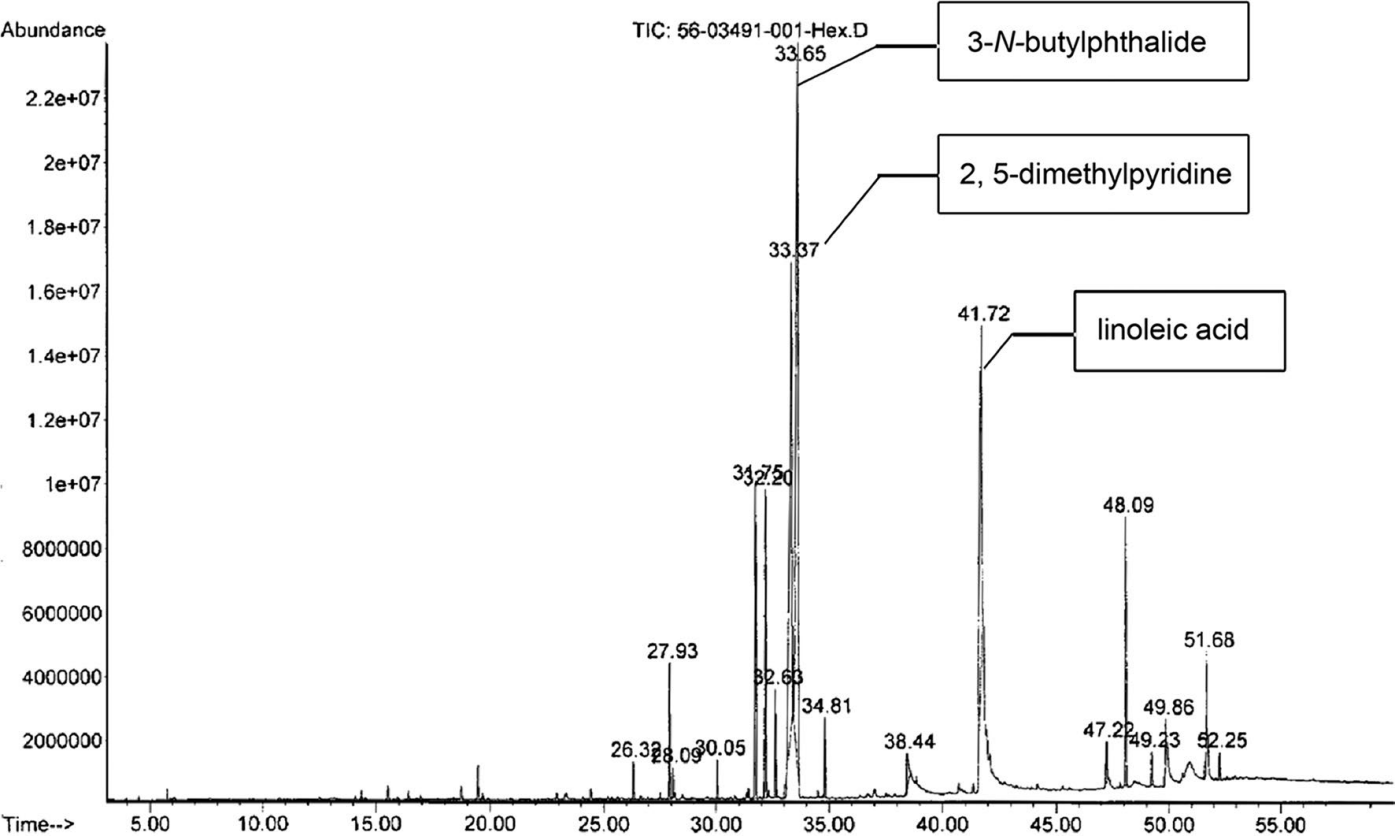
GC/MS Total ion chromatogram of LHE.

Eighteen compounds were derived from LHE, of which the most abundant were 3-*N*-butylphthalide (31.46%), 2, 5-dimethylpyridine (21.94%) and linoleic acid (16.41%), constituting 69.81% of the total non-polar extracts (Fig. 1). The minor constituents of LHE were 4-hydroxyindole (7.05%), butylidene phthalide (6.25%), bis (2-ethylhexyl) phthalate (4.84%) and *β*-selinene (2.41%).

## Discussion

In order to observe repellent responses in different target mosquitoes, evaluations of LHE were carried out against medically important mosquito vectors, including nocturnal (*An. minimus*) and diurnal (*Ae. aegypti*) species, and compared with DEET under laboratory situations. *Ae. aegypti* proved to be more tolerant than *An. minimus* towards both LHE and DEET corresponded to the study of Amer and Mehlhorn [27], who indicated that *Ae. aegypti* was the most aggressive species, with considerably less repellence from plant extracts as well as synthetic substances such as DEET and Icaridin/Saltidin, when compared to *Anopheles stephensi* and *Culex quinquefasciatus*. *Anopheles stephensi* is an urban malaria vector throughout the Indian subcontinent, Middle East and South Asian regions [28, 29], and its lack of resistance to DEET was reported by Klun et al. [30], who also investigated repellency against *An. stephensi* and *Ae. aegypti* of synthetic chemicals, including DEET. Conversely, two populations of *Anopheles albimanus* (El Salvador and Belize strains), which are chief malaria carriers in lowland mid America and the Caribbean [31, 32], were found to be more tolerant than *Ae. aegypti* (red eye Liverpool strain) when treated with DEET and AI3-37220 [33–35]. Although biting tendency differed significantly between the two *An. albimanus* strains, the differences in biting were insignificant. A tolerance to DEET was observed also in the anopheline mosquito, *Anopheles dirus*, which is one of the most efficient malaria vectors in the hilly forested regions of Thailand [6], and it proved to be less sensitive than *Ae. albopictus* [36]. The large variation in biting level among several colonized mosquito species, observed in laboratory repellent tests, was probably due to relative resistance in test mosquitoes, which are present in species rather than population. However, the substantial difference in biting tendency between the two *An. albimanus* strains demonstrated that a large local intraspecific variation among mosquito populations can coexist [35]. Additionally, different susceptibilities to DEET that were found in these sensitive (*An. minimus*, *An. stephensi*) and insensitive (*An. albimanus*, *An. dirus*) *Anopheles* vectors, are evidence that resistance seen in *An. albimanus* and *An. dirus* is not a uniform characteristic in the genus.

The persistent increase in repellency influenced by the synergistic action of vanillin, as reported herein, corresponds with many studies conducted under laboratory conditions, where vanillin enhanced repellency in not only plant-derived products, but also synthetic chemicals such as DEET [27, 37–39]. Due to the renowned ability of vanillin in prolonging protection time, it has been investigated widely as a fixative for mosquito repellents and also inclusion in compositions of various commercial repellents such as Bite Blocker, Bug Spray and Flea & Tick Spray [24, 40–43]. In an effort to enhance the repellent efficacy of LHE, vanillin was utilized in this study as a fixative for reducing the evaporation rates of repellent substances. The results obtained emphasized the benefits of vanillin in optimizing mosquito repellents, and suggested that a promising way to improve the efficacy of repellents is by formulating with appropriate fixatives, such as vanillin. The incorporation of other fragrant fixatives such as fixolide, with promising activity as reported in another study [44], additives and other herbal active ingredients into preparations is considered to be the next step in developing LHE repellent formulations. Other procedures for promoting the performance of natural repellents also should be included, particularly sustained-release technology such as nanoemulsions, nanosuspensions, liposomes and microencapsulation application that provide extended mosquito protection.

Colour changes and varying degrees of repellency recorded among stored LHE samples suggested relatively changeable properties depending on the storing conditions of this product. It was probable that higher temperature induced some reactivity leading to alterations in the character and quality of LHE. Turek and Stintzing [45] also reported that temperature crucially influenced stability of plant-derived products such as essential oil in several respects through oxidative and polymerization processes that resulted in a loss of quality and pharmacological properties. The effect of temperature in reducing repellency of plant-based products, essential oils and solvent extracts against mosquitoes has been reported by many researchers [46, 47]. However, higher temperature may affect the biological activity of stored LHE slightly because its repellency against *Ae. aegypti* was presented for a period of at least 3 months, with insignificantly varied efficacy. Interestingly, LHE samples kept at 4°C, ambient temperature and 45°C for 1 month offered the median complete-protection times of 7.5 (5.0–9.0), 7.25 (5.0–10.5) and 8.0 (4.5–8.5) hours, respectively, which were insignificantly greater than those of the fresh sample (6.5, 5.0–8.0 h). Conversely, most samples stored at each temperature for 2 and 3 months exhibited slightly weaker repellency than the fresh sample and those kept for 1 month. Nevertheless, the stored LHE samples still produced satisfactory protection times (3.5–8.0 h) of more than 2 h, which meets Food and Drug Administration (FDA) requirements for sale in Thailand. To determine the feasibility of such results, analysis of chemical constituents and their alterations in the stored and fresh samples is important for indicating any bioactive substances that are responsible for repellent efficiency. It is interesting that LHE samples kept at ambient temperature for various durations afforded encouraging repellency, which was comparable to that of the fresh sample. This suggests that the product can be placed in a prevailing environment, which makes it convenient and practical to use and maintain.

A limitation in applying plants and plant-derived products, and not standardized ones, is the variable and unstable efficacy depending upon quantity and quality of bioactive constituents, which are mixtures of multiple related compounds. Various factors such as type and stability of plant products, method of extraction, and other plant-related factors including rearing condition (climate and geography), maturation of the harvested plant, plant storage or preservation, and plant preparation, greatly affect the production and alteration of plant components [37, 48–50]. The GC/MS characterization was therefore performed to show the profile of chemicals comprised in LHE, which has no publication on its compositions. A high percentage of phthalides, such as 3-*N*-butylphthalide and butylidene phthalide found in LHE was corresponded to those of many studies previously reported phthalides as the main biological components of *L. sinense* rhizome, besides phenolic acids and polysaccharide [51–55]. Chemical characterization by a similar technique, like GC/MS, of other products from *L. sinense*, such as essential oils, demonstrated variations in type and amount of compound substances. The principal constituents of *L. sinense* oil studied by Huang and Pu [56] were ligustilide (58.00%), 3-butyl phthalide (5.29%) and sabinene (6.08%), whereas those reported by Wang et al. [57] were 5-Oxo-*δ*-4-decahydrobenzindene (50.1%), ligustilide (16.4%) and *β*-phellandrene (7.8%). It was noted that the chemical composition of *L. sinense* rhizome reported in current and previous studies varied qualitatively and quantitatively. The phthalides such as 3-*N*-butylphthalide, butylidene phthalide and ligustilide found in hexane extract or essential oil of *L. sinense* rhizomes also were reported as important constituents in extracted products of celery (*Apium graveolens*) seeds and dong quai (*Angelica sinensis*) rhizomes that offered promising repellency. The hexane-extracted *Apium graveolens* with the main constituents of 3-*N*-butyl-tetrahydrophthalide (92.48%) provided encouraging repellent efficacy when compared to DEET and DEET-based repellents under both laboratory and field conditions [38, 47, 58]. Likewise, Z-ligustilide, the dominant component (61–69%) in *A. sinensis* essential oil, was reported as a potent deterrent against *An. stephensi* and *Ae. aegypti* with higher efficacy than DEET [59]. According to these findings, it is reasonable to assume that phthalides possibly are main active substances responsible for the repellency observed in many plant-derived products, including LHE. This study, however, did not clarify the potential repellent activity of phthalides against mosquitoes because these compounds could not be separated by GC, due to their instability in the GC column.

It is accepted generally that laboratory study is essential in obtaining the preliminary assessment of repellent property and mosquito sensitivity from this situation, which can be an initial indicator of repellency against mosquitoes. Nevertheless, the protective effect of repellents against the mosquito model may not assure success against other species or even the same species under different circumstances. Repellent that significantly repels mosquitoes in one population at one time may be more or less effective against the same species at a different locality or time [35]. Laboratory and field evaluations of synthetic substances, including DEET, CIC-4 and AI3-37220, against *An. dirus* in Thailand, demonstrated a different status of these repellents [60]. While *An. dirus* tested under laboratory conditions was found to be more sensitive to CIC-4 than either DEET or AI3-37220, subsequent field study in Chanthaburi province, eastern Thailand, revealed that protection provided by AI3-37220 (>95% protection for 4 h) was significantly better than either DEET or CIC-4 (<95% for 2 h), and there was no significant difference between DEET and CIC-4. However, another field trial in Sisaket province, northeastern Thailand, discovered that repellents containing 33, 50 and 70% DEET provided complete protection for up to 6 h against primarily *Culex vishnui* and *An*. *dirus* [61]. Correspondingly, results obtained from laboratory testing with *Anophelesfarauti*, a vector of malaria in the southwest Pacific region [62], contrasted to those of the field evaluation. While concentrations of 5–50% DEET provided up to 130 min protection against *An*. *farauti* under laboratory conditions, only 25% DEET afforded greater than 95% protection for five and at least 4 h against field *An*. *farauti* in northern Queensland, Australia, [63] and wild *Anopheles* spp. in Lae, Papua New Guinea, respectively [64]. It can be stated regarding this that the repellent response in mosquitoes varies in either species or population. Consequently, it is important to conduct both laboratory and field trials against a variety of potential vector species; make preliminary assessments in laboratory and ultimately field experiments to obtain information on repellent effectiveness against natural pests and vector species. Therefore, repellent evaluations of LHE against various mosquito species, particularly insecticide-resistant strains and natural mosquito populations in Chiang Mai province, are being studied further. As LHE has proven repellent efficacy against different mosquito vectors and is physically and biologically stable, with no irritant side effects, it qualifies as a new natural alternative to DEET, or an additional weapon for use together with other chemicals/measures for integrated vector control.

## Conclusions

The remarkable repellency of LHE, which is comparable to DEET, has proved its promised potential for development as an alternative repellent against mosquito vectors, particularly *An. minimus* and *Ae. aegypti*. Further action is being considered, with some already in progress on the isolation and identification of active ingredients, improved formulation by simple and advanced techniques, and repellent investigation against other mosquitoes of medical importance under laboratory and field conditions. All these results are useful and warrant promoting utilization and development of potentially alternative repellents, based on bioactive substances from indigenous herbal resources.
